# Using E‐portfolios to Identify Threshold Concepts in Removable Prosthodontics

**DOI:** 10.1002/jdd.13943

**Published:** 2025-06-25

**Authors:** Edward Waters, Delyse Leadbeatter

**Affiliations:** ^1^ Sydney Dental School Faculty of Medicine and Health University of Sydney Surry Hills Australia; ^2^ School of Medicine University of Notre Dame Australia Darlinghurst Australia

**Keywords:** Curriculum, dental, education, graduate, prosthodontics, qualitative research, thinking

## Abstract

**Introduction:**

Training in removable prosthodontics traditionally includes a preclinical laboratory component that precedes clinical exposure. Students struggle to relate the laboratory component to clinical work, because successful learning in removable prosthodontics involves many threshold concepts, some of which have been identified previously. No existing studies consider whether e‐portfolios can help educators and learners identify threshold concepts.

**Methods:**

Students at a single dental school in Australia complied an e‐portfolio detailing their learning experiences in a preclinical removable prosthodontics laboratory continuum in 2023. Their responses were analyzed using deductive qualitative analysis.

**Results:**

Two themes were identified (functional occlusion and functional records). Student responses associated each theme with transformative insights, knowledge integration, and bounded professional practice (three features of threshold concepts).

**Conclusion:**

Analysis of e‐portfolios may be used to identify threshold concepts in dental education. Functional occlusion and records, two thematic constructs identified in our analysis, may be threshold concepts in removable prosthodontics.

## 1 Introduction

Training in removable prosthodontics (dentures) is an essential component of dental education [1]. The clinical skills and knowledge required to successfully provide removable dentures are essential foundations for providing implant‐supported restorative options, and for aesthetically focused treatment planning [2]. Aside from developing foundational skills that transfer to other areas of restorative dentistry, removable prosthodontics remain an important restorative option in their own right. Many patients prefer to have edentulism managed using removable dentures or are unsuitable for alternative options such as implants or bridgework. Thus, removable prosthodontics remains an essential component of dental education in the 21st century [3].

Despite the unquestionable importance of skills in removable prosthodontics in contemporary dentistry, it is not clear that our educational practices are optimal. Traditionally, dental students have developed preliminary knowledge and skills in removable prosthodontics through a course of lectures and seminars, combined with undertaking technical tasks that underpin the process of denture fabrication in a dental laboratory [4]. Dental students struggle to relate this didactic and technical instruction to clinical scenarios because, in the traditional removable prosthodontic education continuum, they receive this training months or years before they ever see an edentulous patient [4, 5]. Reflecting this, a recent study found that senior dental students in the United Kingdom thought that laboratory and technical training was over‐emphasized in removable prosthodontics education, and clinical activities were superior at improving their confidence with this treatment option [6]. Reductions in laboratory time have been discussed in dental education literature for over 30 years [7], but preclinical laboratory training remains part of most curricula. The question of how to assist students to understand how these skills relate to patient care, which they will not be involved in until later on in their courses, remains a challenge in dental education.

Several authors have re‐framed this problem in terms of threshold concepts [1, 5]. Threshold concepts are milestones in learning that result in transformations of the way that students understand and practice [8, 9]. Examples of threshold concepts in removable prosthodontics are:

1. “product visualization”—needing to visualize what a denture is and does before being able to take jaw relation records to make one [9] and that
2. “aligning patient and practitioner expectations” is essential for successful prosthodontic treatment [5].

However, these observations relate to the need to navigate threshold concepts in the clinical setting—little work has been done on how to navigate threshold concepts in the laboratory setting, which seems an even more significant task. When even the clinical steps of taking jaw relations are difficult for students to relate to providing a satisfactory removable restoration [9], it is easy to see how students struggle to relate laboratory work to clinical practice when they have never even seen an edentulous patient [4]. One of the authors, reflecting on their experience of this training, noted their bewilderment when first being confronted with an edentulous cast in a laboratory exercise. At that point in their education, they remembered, “I don't think I even knew it was possible for someone to be edentulous.” This quote poignantly illustrates the significance of threshold conceptual knowledge. If a provider does not understand the restorative need, they cannot envision the prosthetic requirements—so how can they understand how a laboratory procedure contributes to achieving a satisfactory prosthetic outcome?‘’

The purpose of this study was to understand whether laboratory e‐portfolios assist dental students in navigating threshold concepts in removable prosthodontics. We surmised that if they were effective learning tools, evidence of students identifying threshold concepts would be discoverable in their e‐portfolio entries. Given the limited number of threshold concepts identified thus far in the removable prosthodontics education literature, we anticipated that the concepts identified by our students might also be previously undocumented. We tested these hypotheses using deductive qualitative analysis (DQA).

## 2 Materials and Methods

### 2.1 Ethical Issues

The study received ethics approval from the University's Human Research Ethics Committee, approval number 2023/HE000571.

### 2.2 Data Sources

Data for the project formed part of e‐portfolios submitted by first‐year dental students at our institution undertaking a preclinical laboratory continuum of denture fabrication procedures. They received instruction via a proprietary laboratory manual as well as by practical demonstrations and evaluation of work by laboratory technical staff. Outside of this continuum, students received didactic lectures on clinical and theoretical aspects of denture treatment.

**FIGURE 1:**
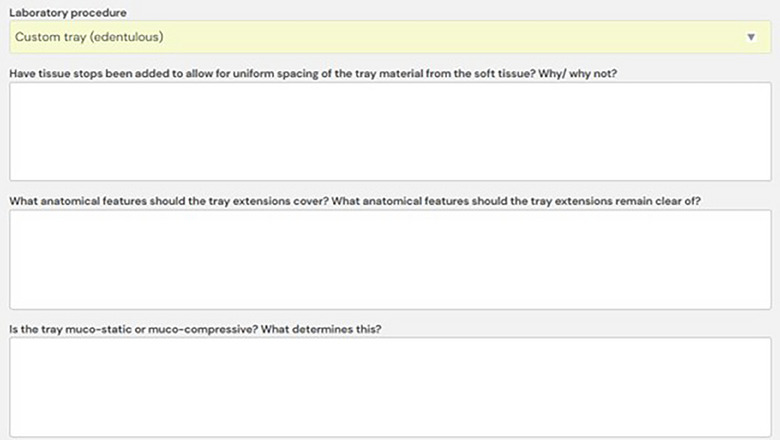
Example of reflective prompts provided to students in the E‐portfolio form relating to the fabrication of custom trays.

In 2023, our School implemented a new curriculum based on programmatic assessment [10], featuring the use of e‐portfolios to facilitate work‐based learning and reflective practice [11]. As an initiative to facilitate learning removable prosthodontics in the new curriculum, a set of reflective forms was created on the e‐portfolio system (risr/advance—https://risr.global/advance). These forms required students to reflect on dental laboratory procedures. For example, the first form asked the student to reflect on the steps involved in taking alginate impressions on a phantom head and pouring them up. Subsequent forms covered making maxillary wax rims, mandibular wax rims, custom impression trays, articulating casts, and setting teeth. An example of the prompts given for the fabrication of custom trays is shown in Figure 1.

To facilitate the use of the e‐portfolio forms as a resource to draw on throughout their removable prosthodontics training, students had the ability to upload images of their work, drawings, etc., to the form. The forms were not marked or assessed by staff, and were also optional—students could use them or not in self‐directed learning if they found them useful.

### 2.3 Data Collection

A purposive sampling strategy was employed. All students who completed the e‐portfolio of removable prosthodontics forms in 2023 were eligible to participate in the study. Students were asked to opt in to have their e‐portfolios analyzed for this project. The approach was via an email from a nonacademic member of staff who was not involved in the project. The recruitment email contained a link to an online consent form. A reminder email was sent four weeks after the initial email. The investigators received notifications when any student completed the consent form to enroll in the study. All completed e‐portfolio forms were downloaded for analysis.

Ten students enrolled in the study, each of whose e‐portfolios consisted of the six forms described above (60 data points analyzed in total). The adequacy of this sample was assessed by testing for theoretical saturation, as described below.

### 2.4 Data Analysis

This study employed DQA, one of a family of methods developed by the Chicago School of sociology in the mid‐20th century [12]. Qualitative research is generally associated with inductive “bottom‐up” approaches to developing theory, of which grounded theory (GT) is the example *par excellence*. Yet, GT and DQA in fact constitute a deductive‐inductive continuum of qualitative research methods developed by the Chicago school. In deductive qualitative research, a priori hypotheses are not simply permissible, but essential [13].

In the current study, our data analysis was not theoretically agnostic, so a GT approach was not appropriate. As described above, we held a priori hypotheses that threshold concepts were involved in learning removable prosthodontics, and that our students’ articulation of existing and new threshold concepts might be discoverable in our data. Thus, this research demanded a deductive rather than inductive methodological approach, and hence we decided to employ DQA.

**FIGURE 2:**
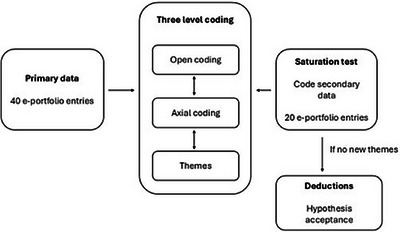
Graphical representation of the data analysis process.

The rigor of DQA is built on proposing, analyzing, and modifying hypotheses, based on data (if not A, then B). In DQA, a priori hypotheses are affirmed in the presence of confirmatory data points, called positive cases in the literature. within DQA, positive cases are only considered to conditionally support a priori hypotheses, as new data may arise that negates or modifies our understanding [13]. Because it can be iteratively updated, DQA has similarities to constructivist GT [14]. Methods of data analysis used in DQA are therefore similar to those used in GT (constant comparison and coding) [14]. For coding, we grouped the forms into two groups. The first four of the six forms (primary data) provided by each student (primary impressions, maxillary wax rim, mandibular occlusal rim, custom impression tray) were used for primary analysis. The remaining two forms (articulation of casts and setting teeth—secondary data) were used to assess theoretical saturation. When theoretical saturation was assessed in the positive, deductions were made in favor of the hypothesis that threshold concepts occur in learning removable prosthodontics. A graphical representation of the data aggregation and coding processes is provided in Figure 2.

The primary analysis focused on identifying positive cases (e‐portfolio contains evidence of threshold concepts). This was undertaken by reviewing the primary data and highlighting statements that comprised either complete or logical syllogisms, or contained incomplete syllogisms (articulation of premises without conclusions) [14]. These highlighted passages were used to develop open codes, which were subsequently grouped into axial codes and themes via a process of constant comparison. The coding process was deemed to be complete when theoretical saturation was obtained. This was assessed by applying the same analytical process to the secondary data and assessing whether any new open codes, axial codes, or themes were identified (see Figure 2).

Coding was done by a solo researcher (E. W.). This decision was driven by the rapid time in which the study needed to be completed (a single semester, in order to evaluate the e‐portfolio tool for inclusion in the curriculum in subsequent years) and by the relatively narrow nature of the question. When the study focus is restricted and time‐bound, analysis by solo‐researchers can represent best practice [15]. Peer debriefing with the senior researcher (D. L.) was used to assess external validity and the reliability of the coding process. This is a recognized approach to enhancing reliability in qualitative research where the use of multiple coders is not feasible or appropriate [16].

## 3 Results

**FIGURE 3:**
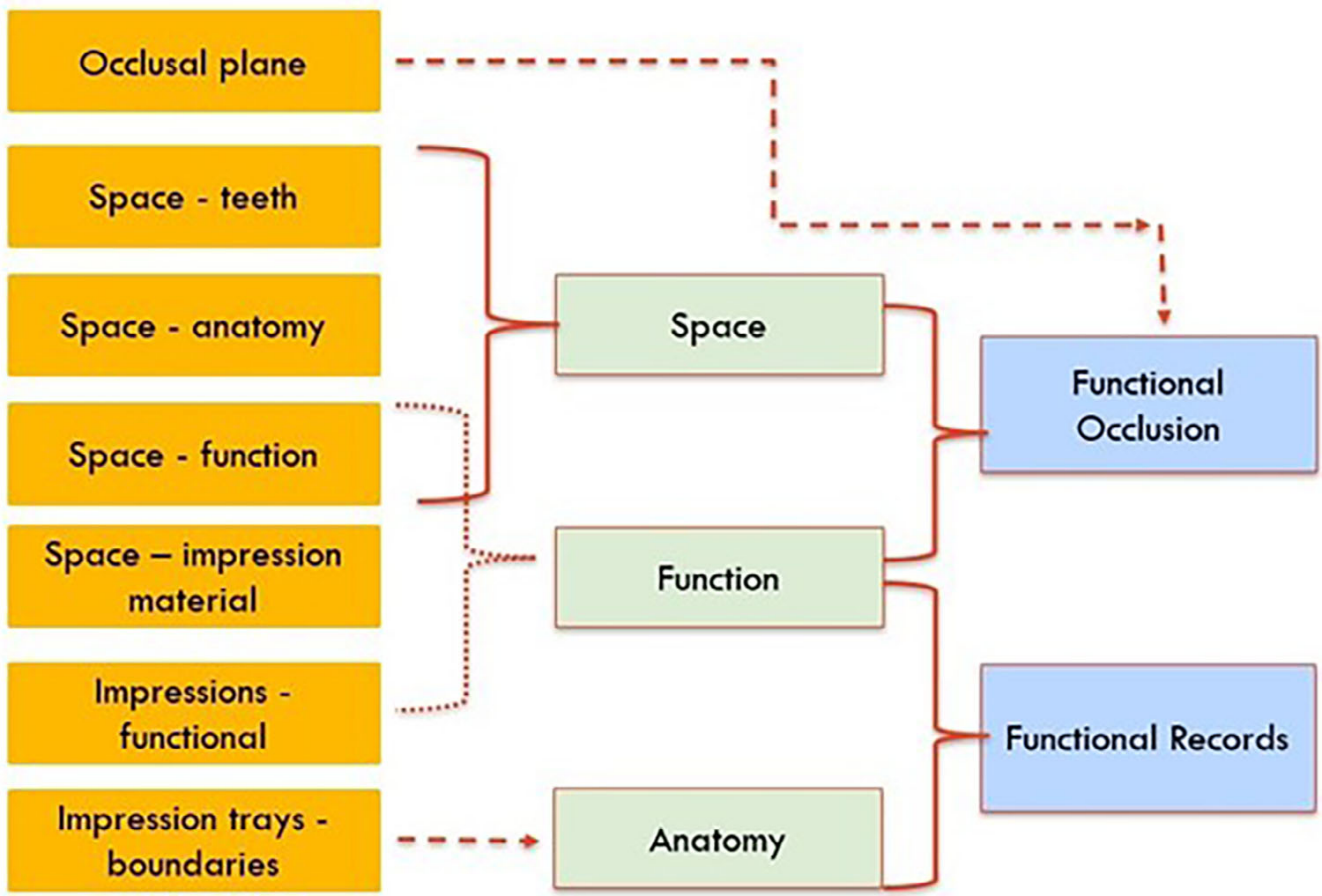
Open codes (orange), axial codes (green), and themes (blue).

Seven open codes were identified during the analysis of the primary data, as shown in Figure 3. Excerpts from the primary data that were associated with these open codes are given in Table 1. Open‐coded excerpts were grouped into three axial codes, and two themes were identified from the process of axial coding—*functional occlusion* and *functional records*.

**TABLE 1:** Examples of open codes attributed to passages within the primary data.

	Excerpt	Open code
(a)	Because otherwise, the maxillary occlusal rim would not accommodate the retromolar pad on the mandibular rim when the jaw closes	Space—anatomy
(b)	The patient's tongue will be crowded/ restricted	Space—anatomy
(c)	The occlusal surface should be 8–10 mm. If the rim is too thin or too wide, it might not occlude properly with the maxillary rim when taking a bite, resulting in an inaccurate measure of the patient's maxillo‐mandibular relationships	Occlusal plane
(d)	Tissue stops are used when taking the selective pressure technique, which requires a space design for the special tray (spaced special tray)	Space (impression material)

All excerpts in the secondary data were related to two open codes—space and the occlusal plane—and associated with the single theme of *functional occlusion*. Exemplar excerpts from the secondary data, along with their open and axial codes, are given in Table 2. As no new themes were identified from the secondary data, theoretical saturation was assumed to have been reached using the available sample.

**TABLE 2:** Examples of open codes, axial codes, and themes attributed to passages within the secondary data.

	Excerpt	Open code	Axial code	Theme
(a)	Dentures with balanced occlusion enable the patient to use them comfortably during all activities	Occlusal plane	Function	Functional occlusion
(b)	Both the curve of Wilson and Spee relate to the natural curves of the dentition	Occlusal plane	−	Functional occlusion
(c)	Curve of Spee is the anteroposterior curve, curving in the media plane that allows the protrusive dislocation of the posterior teeth	Occlusal plane	Function	Functional occlusion
(d)	The maxillary second molar will be off the occlusal plane and follow the curve of Spee	Occlusal plane	−	Functional occlusion
(e)	The curve of Wilson is the mediolateral curve that permits lateral mandibular excursions free from posterior interference	Occlusal plane	Function	Functional occlusion
(f)	There needs to be two posterior points of contact in the occlusal plane and one anteriorly	Occlusal plane	−	Functional occlusion
(g)	The curve allows the occlusal forces to be evenly distributed across the dental arches during functional movements	Space—function	Space	Functional occlusion
(h)	Curve of Wilson: It represents the mediolateral (side to side) curvature of the occlusal plane. This curve helps accommodate the angulations of the posterior teeth	Space—teeth	Space	Functional occlusion

The two themes identified in this study—*functional occlusion* and *functional records*—are presented as new threshold concepts in removable prosthodontic education. This claim is justified by our deductive methodology. The coded data underpinning the identification of these themes deductively attributes three features of threshold concepts to each theme. The concepts thematically classified under *functional occlusion* and *functional records* can be considered transformative, integrative, and bounded (see Tables 3 and 4).

**TABLE 3:** Relationship between the two themes identified and some features of threshold concepts [15].

Attribute	Theme—functional records	Theme—functional occlusion
Transformative	Requirement to capture tissues under function (selective pressure) to create functional denture—see Table 1(d)	Occlusal plane—important not just for static relationships, but for function—see Table 2(a,d,f,h)
Integrative	Integrates concepts around impression materials, armamentarium (impression trays and rims), and anatomy—see Table 1(a,d)	Students begin to integrate laboratory work with clinical performance—see Table 2(a)
Bounded	It may appear limited to denture fabrication—can students relate it to other procedures?	Establishes denture occlusion as a specific area of knowledge

**TABLE 4:** Some features of threshold concepts (adapted from Meyer and Land) [15].

Threshold concepts are likely to be: Transformative Irreversible Integrative Bounded Potentially troublesome

## 4 Discussion

This study builds on previous work about how students use threshold concepts to integrate learning in removable prosthodontics [1, 5, 9, 16]. On the basis of analysis of student work, it proposes two new threshold concepts that are important in learning removable prosthodontics—*functional occlusion* and *functional records*.

Our claim that these two themes are threshold concepts is partly justified by our deductive methodological process, and partly by the extent to which the textual excerpts presented demonstrate that these themes exhibit a number of the features of threshold concepts, as enumerated by Meyer and Land (see Table 4) [17]. Specifically, there is evidence that these themes are associated with *transformative* insights, knowledge *integration*, and establish *bounded* categories of knowing and understanding that assist with further learning (see Table 3).

First, the *transformative* nature of knowledge about functional record‐taking and occlusion is deduced from how these relate to product visualization, an established threshold concept in removable prosthodontics [9]. Our data and analysis suggest that students can use a preclinical laboratory skills continuum to not only visualize what they will produce (a denture), but also whether it will be functionally acceptable (see Table 3). Our students’ understanding that the properties of the final denture are affected by record taking and laboratory work suggests a transformation from knowing *how* something is done to *why* it is clinically important.

Second, our students’ writing about these two themes provides evidence of knowledge *integration*. Some excerpts provide evidence that students were beginning to integrate knowledge in their efforts to understand how functional records are obtained and functional occlusion is developed in the dental laboratory (see Table 3). The excerpts demonstrate the students integrating their learning in two specific ways, which have been described in the literature as “widening the lens” (Table 1a,d) and discerning “purposeful actions” (Table 2a) [18]. The latter type of learning integration is facilitated by the transformative nature of threshold concepts in removable prosthodontics, as understanding why things are done is essential to being able to implement them clinically [9].

Finally, our identified threshold concepts of functional record taking and occlusion are likely *bounded* (restricted to removable prosthetics), albeit that these boundaries may themselves be transformative in later helping students to understand how record‐taking and occlusion in complete dentures differs from that in other areas of dentistry (e.g., implants and temporo‐mandibular disorders). It is also reasonable to deduce that knowledge of the identified threshold concepts is at least partly *irreversible*, given that students will have had much more limited understanding of denture fabrication prior to entering the course, and cannot return to a denture‐naive state (although this is not included as a result in Table 3 since no specific excerpts from our students discussed knowledge retention).

Beyond proposing new threshold concepts in removable prosthodontics, this study also makes new contributions to the literature on e‐portfolios. Specifically, it demonstrates how e‐portfolios can be used to study threshold concepts. In the past decade, e‐portfolios have been used extensively in education [11], including in dentistry [19, 20]. To our knowledge, however, this study is the first that describes their use in preclinical removable prosthodontics education in a dental school. Likewise, there is only a limited literature that links threshold concepts to e‐portfolios, and this only describes a set of threshold concepts that should be used in designing e‐portfolios [21, 22]. There are no previous studies that use e‐portfolios as a data source to understand how students navigate threshold concepts or to identify new ones. By using data from e‐portfolios to propose two new concepts in removable prosthodontics, we have shown that they can potentially be a valuable data source for identifying these. We cannot assert that e‐portfolios help students to navigate threshold concepts, because we are not able to compare students who did and did not use the e‐portfolio. However, our study does further contribute to the body of evidence that suggests that e‐portfolios are useful to bridge the gap between learning and practice [23, 24].

An incidental finding in this study related to how students completed the e‐portfolio forms. Unexpectedly, the dates at which students completed forms were not chronologically related to the times at which they participated in the laboratory sessions. Within our sample, some students completed e‐portfolio forms asynchronously. For example, some completed the e‐portfolio form on articulating study casts after the form on setting teeth. Likewise, some updated the form on primary impressions after finalizing other forms. This suggests that at least some students iteratively returned to these forms, either to assist in their study process or to update their entries after additional learning and reflection on the laboratory tasks. This unexpected finding suggests that the capacity to iteratively engage with e‐portfolio content may be useful in student learning, and this should be studied further.

There are some limitations to this study. Students self‐selected, and the final number of portfolios examined was therefore small (although the number of individual portfolio *entries* examined was relatively greater). This is commonly reported as a limitation of qualitative studies [25]. It might be argued that the sample of portfolios examined might not be representative, limiting the generalizability of our findings. However, qualitative research need not claim to be generalizable [24], preferring instead to focus on whether it is trustworthy [26, 27]. Trustworthiness is enhanced by robust theoretical frameworks, appropriate study design, and transparency rather than by large sample size [25, 28]. Our transparent account of our deductive inferential process and our methods of data collection and analysis are presented as ways of overcoming the limitations of this study and increasing its trustworthiness [28].

Another limitation is that students were enrolled in a preclinical laboratory curriculum, so we cannot assert that the learning evidenced in the e‐portfolios will translate to their clinical performance. This will need to be assessed by an additional research project once the students enter the clinical years of the program. Nonetheless, this study has importance for understanding and enhancing learning in preclinical dental laboratory training. It may also be useful for administrators of dental laboratory technician programs who are exploring ways to develop greater clinical understanding amongst the future dental technology workforce. The findings of this study suggest that threshold concept acquisition should inform the development of dental laboratory curricula in both dental and technician programs.

## 5 Conclusion

Functional occlusion and functional records may be important threshold concepts that assist dental students undertaking a preclinical removable prosthodontics continuum to subsequently apply their learning to clinical practice. These concepts were identified via an analysis of e‐portfolio entries by dental students from a single university, recounting their learning within the removable prosthodontics laboratory setting. The results of this study build on previous literature that equates learning in removable prosthodontics with negotiating threshold concepts. More innovatively, it establishes an analysis of e‐portfolio data as a method for identifying threshold concepts.

## Conflicts of Interest

The authors declare no conflicts of interest.
